# Red Blood Cell Eicosapentaenoic Acid Inversely Relates to MRI-Assessed Carotid Plaque Lipid Core Burden in Elders at High Cardiovascular Risk

**DOI:** 10.3390/nu9091036

**Published:** 2017-09-20

**Authors:** Núria Bargalló, Rosa Gilabert, Edwin-Saúl Romero-Mamani, Montserrat Cofán, Philip C. Calder, Montserrat Fitó, Dolores Corella, Jordi Salas-Salvadó, Miguel Ruiz-Canela, Ramon Estruch, Emilio Ros, Aleix Sala-Vila

**Affiliations:** 1 Radiology Department, Clinical Diagnostic Imaging Centre, Institut d’Investigacions Biomèdiques August i Sunyer, Hospital Clínic, 08036 Barcelona, Spain; bargalloclinic.ub.es (N.B.); gilabert@clinic.ub.es (R.G.); 2 Magnetic Resonance Image Core Facility, Institut d’Investigacions Biomèdiques August i Sunyer, 08036 Barcelona, Spain; 3 Department of Internal Medicine, Institut d’Investigacions Biomèdiques August i Sunyer, Hospital Clínic, 08036 Barcelona, Spain; eromero@hotmail.com (E.-S.R.-M.); restruch@clinic.cat (R.E.); 4 Ciber Fisiopatología de la Obesidad y Nutrición , Instituto de Salud Carlos III, 28039 Madrid, Spain; mcofan@clinic.cat (M.C.); mfito@imim.es (M.F.), dolores.corella@uv.es (D.C.); jordi.salas@urv.cat (J.S.-S.); mcanela@unav.es (M.R.-C.); eros@clinic.ub.es (E.R.); 5 Lipid Clinic, Department of Endocrinology and Nutrition, Institut d’Investigacions Biomèdiques August i Sunyer, Hospital Clínic, 08036 Barcelona, Spain; 6 Human Development and Health Academic Unit, University of Southampton, Southampton SO16 6YD, UK; P.C.Calder@soton.ac.uk; 7 NIHR Southampton Biomedical Research Centre, University Hospital Southampton NHS Fundation Trust, Southampton SO16 6YD, UK; 8 Institut Hospital del Mar d'Investigacions Mèdiques, Barcelona Biomedical Research Park, 08003 Barcelona, Spain; 9 Department of Preventive Medicine, University of Valencia, 46010 Valencia, Spain; 10 Human Nutrition Department, Hospital Universitari Sant Joan, Institut d'Investigació Sanitaria Pere Virgili, Universitat Rovira i Virgili, 43201 Reus, Spain; 11 Department of Preventive Medicine and Public Health, University of Navarra, 31080 Pamplona, Spain

**Keywords:** atherosclerosis, diet, fish, imaging, omega-3

## Abstract

Supplemental marine omega-3 eicosapentaenoic acid (EPA) has an anti-atherosclerotic effect. Clinical research on EPA supplied by the regular diet and atherosclerosis is scarce. In the framework of the PREvención con DIeta MEDiterránea (PREDIMED) trial, we conducted a cross-sectional study in 161 older individuals at high vascular risk grouped into different stages of carotid atherosclerosis severity, including those without ultrasound-detected atheroma plaque (*n* = 38), with plaques <2.0 mm thick (*n* = 65), and with plaques ≥2.0 mm (*n* = 79). The latter were asked to undergo contrast-enhanced 3T magnetic resonance imaging (MRI) and were subsequently grouped into absence (*n* = 31) or presence (*n* = 27) of MRI-detectable plaque lipid, a main feature of unstable atheroma plaques. We determined the red blood cell (RBC) proportion of EPA (a valid marker of long-term EPA intake) at enrolment by gas chromatography. In multivariate models, EPA related inversely to MRI-assessed plaque lipid volume, but not to maximum intima-media thickness of internal carotid artery, plaque burden, or MRI-assessed normalized wall index. The inverse association between EPA and plaque lipid content in patients with advanced atherosclerosis supports the notion that this fatty acid might improve cardiovascular health through stabilization of advanced atheroma plaques.

## 1. Introduction

Atherosclerosis is the harbinger of coronary heart disease (CHD), the leading cause of mortality worldwide [1]. Because of the systemic nature of atherosclerosis, the presence of atheroma plaques anywhere in the arterial tree is indicative of advanced disease in the coronary vascular beds. This evidence prompted the use of noninvasive imaging techniques to assess the atherosclerotic burden in easily accessible carotid arteries. While ultrasound-detected carotid plaque improves the risk prediction of future cardiovascular events [2], carotid magnetic resonance imaging (MRI) allows to reproducibly quantify major plaque components indicative of plaque vulnerability, such as the presence of a lipid-rich core [3].

There is a large body of observational evidence on the primary prevention of CHD, in particularly sudden cardiac death, by fish-derived omega-3 fatty acids, mainly docosahexaenoic (C22:6*n*-3, DHA) and eicosapentaenoic (C20:5*n*-3, EPA) acids [4]. The anti-atherosclerotic properties of the latter (recently reviewed in [5]) contributes to its cardioprotective effect. However, this notion relies mostly on the results of trials of supplementation with either pure EPA or fish oils, while studies on EPA supplied by the usual diet are scarce. Given the difficulties of accurately measuring long-term fat intake from diet records, the optimal approach to obtain reliable data is by determining the fatty acid composition of adipose tissue, although circulating fatty acids are a convenient and accepted alternative [6]. The turnover of red blood cells (RBCs) (120-day lifespan) makes RBCs suitable for objective assessment of the omega-3 fatty acid status [7].

In line with this, in a recent comprehensive review of the relationship between blood levels of omega-3 fatty acids and cardiovascular risk, the authors concluded that “evidence exists supportive of the concept that the higher the blood level, the greater the effect on atherosclerosis and, owing to individual variability, the blood levels of omega-3 fatty acids may be better markers of cardiovascular risk/benefit than simple assignment to a fixed dose of omega-3 supplementation” [8]. We hypothesized that, in individuals at high vascular risk but free from cardiovascular disease, circulating EPA would inversely relate to the atherosclerotic burden across the spectrum of the disease. To address this issue, in 161 individuals grouped into different stages of carotid atherosclerosis severity, ranging from absence of atheroma plaque to the presence of a lipid-rich plaque core, we determined the proportion of EPA (and other selected omega-3 fatty acids) in RBC membranes and investigated its association with various carotid imaging variables.

## 2. Material and Methods

### 2.1. Setting

This cross-sectional study was conducted within the frame of the PREvención con DIeta MEDiterránea (PREDIMED) trial [9] (http://www.predimed.es). Participants were men aged 55 to 80 years and women aged 60 to 80 years. Criteria for eligibility were the presence of either type 2 diabetes or at least three of the following cardiovascular risk factors: current smoking, hypertension, dyslipidemia, overweight or obesity, and family history of early-onset CHD. For this analysis we selected 283 consecutive participants recruited in the Barcelona-North site between 2008 and 2010. At enrolment, study subjects underwent blood sampling and were offered carotid ultrasound and, when sizable plaques were detected, carotid MRI examination. The study protocol was conducted according to the guidelines laid down in the Declaration of Helsinki and all procedures were approved by the institutional review board of Hospital Clínic, Barcelona, Spain (reference 1244 from the Clinical Research Ethics Committee). Written informed consent was obtained from all study participants.

### 2.2. Assessment of Risk Factors and Dietary Habits

Participants were considered as diabetic, hyperlipidemic or hypertensive if they had a previous diagnosis of these conditions and/or they were treated with antidiabetic, cholesterol-lowering, or antihypertensive agents, respectively. Smoking status was categorized into never, current or past smoking according to self-reports. Physical activity was determined with a validated Spanish version of the Minnesota Leisure-Time Physical Activity questionnaire. Height, weight and waist circumference were measured with standard methods. Trained personnel measured systolic and diastolic blood pressure in triplicate with a validated semi-automatic oscillometer (Omron HEM-705CP; Hoofddorp, The Netherlands). A trained dietitian obtained data on dietary habits of participants in a face-to-face interview using a validated 137-item food frequency questionnaire including 8 seafood-related items [10]. Nutrient intakes were computed using Spanish food composition tables [11] and were adjusted for energy intake by the residual method [12]. The validation of the food frequency questionnaire against 3-day food records showed energy-adjusted intraclass correlation coefficients of 0.51 for EPA + DHA (*P* < 0.001).

### 2.3. Cell Membrane Fatty Acid Analysis

Overnight fasting (>10 h) blood samples were drawn and stored at −80 °C until analysis. The fatty acid profile was determined as described [13]. In brief, cells were hemolysed, spun, and the pellet (>99% RBC membranes) was dissolved in 1 mL BF_3_ methanol solution and transferred to a screw-cap test-tube, which was heated for 10 min at 100 °C to hydrolyse and methylate glycerophospholipid fatty acids. After cooling, fatty acid methyl esters were isolated by adding 1 mL of a saturated K_2_CO_3_ solution and 300 μL of *n*-hexane. An aliquot of the upper (hexane) layer was transferred into an automatic injector vial equipped with a 300 μL volume adapter. Fatty acid methyl esters were separated by gas chromatography using an Agilent HP 7890 Gas Chromatograph equipped with a 30 m × 0.25 μm × 0.25 mm SupraWAX-280 capillary column (Teknokroma, Barcelona, Spain), an autosampler, and a flame ionization detector. The amount of each fatty acid (EPA, DHA and C18:3*n*-3 (alpha-linolenic acid, ALA, the plant omega-3)) is expressed as a percentage of the total identified fatty acids in the sample. The omega-3 index was calculated as the sum of percentages of EPA + DHA.

### 2.4. Carotid Ultrasonography

B-mode ultrasound imaging of the carotid arteries was performed with an ultrasound apparatus (Sequoia Acuson; Siemens, Erlangen, Germany) equipped with a multi-frequency transducer (7–10 MHz) and electrocardiogram synchronization. All procedures were performed by a certified radiologist (RG) who was blinded to clinical information. A standardized imaging protocol was used for intima-media thickness (IMT) measurements as described in detail elsewhere [14]. IMT was defined as the average of multiple distance readings between the far-wall lumen–intima and media–adventicia interfaces taken bilaterally at the common carotid artery 1 cm prebifurcaction, bifurcation, and the internal carotid artery (ICA) 1 cm after the flow divider. Plaques were sought by using B-mode and colour Doppler examinations in both longitudinal and transverse planes to take into consideration circumferential asymmetry, and were defined as focal intrusions into the lumen ≥1.2 mm thick. IMT and maximum plaque height were measured offline in predefined segments of the arterial wall. Plaque burden was calculated as the sum of maximum heights of all plaques.

### 2.5. Carotid MRI

Individuals with plaques ≥2 mm were asked to undergo carotid MRI, which was performed with a commercial 3T whole-body scanner (MAGNETOM Trio; Siemens, Erlangen, Germany) equipped with a dedicated 4-channel phased array carotid coil (Machnet BV, The Netherlands). A normal 3-plane localizer was followed by a vascular localizer to identify the carotid bifurcation. Fourteen axially oriented imaging slices centered at the carotid bifurcation and covering ≈24 mm of the internal carotid, bifurcation and common carotid were obtained. Black-blood T1-weighted , T2-weighted, and proton density-weighted data sets were obtained based on an inflow/outflow saturation band (IOSB) sequence as described by Koktzoglou and collaborators [15]. For 3D imaging of plaque morphology, a T2W Single Slab 3D TSE with Variable Flip Angle Distribution and High Isotropic Resolution (3D SPACE) sequence was used. A high-resolution contrast-enhanced MRI was performed to determine percent stenosis and a delayed enhancement technique was used to see contrast enhancement in plaques. After acquisition, magnetic resonance images were coded and saved on CD and in picture archiving and communication system. For each slice, a certified radiologist (NB) drew contours of the outer wall, lumen and lipid core, which was identified using the criteria previously described in a histologically validated technique [16]. Vessel wall area (outer wall minus lumen) and lipid area were quantified using a customized software program (Vessel Mass Software, Leiden University Medical Center, The Netherlands). Normalized wall index was calculated by dividing the vessel wall area by the total vessel area. Plaque lipid volume was obtained by summing respective area measurements for each artery and multiplying by slice thickness (2 mm).

### 2.6. Statistical Analyses

**Figure 1:**
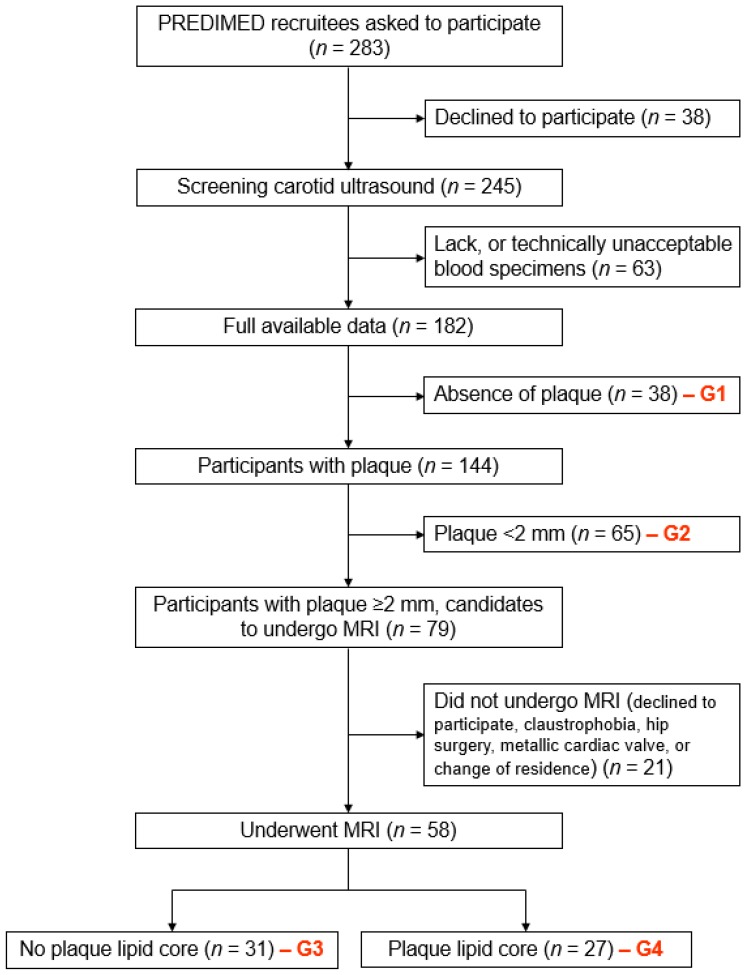
Flow-chart of the study. “PREDIMED” denotes “PREvención con DIeta MEDiterranea”; “G” denotes group; “MRI” denotes “magnetic resonance imaging”.

We asked 283 PREDIMED recruitees to participate in the study (Figure 1). Screening carotid ultrasound was performed in 245 candidates. From this group we excluded 63 participants because of a lack of or technically unacceptable blood specimens. Thirty-eight out of 182 participants with full data available (21%) had no plaque (Group 1), while 65 (35%) had plaques with thickness <2 mm (Group 2). Among those with plaques ≥2 mm, 21 subjects did not undergo MRI for several reasons (declined to participate, claustrophobia, hip surgery, metallic cardiac valve, or change of residence). As a result, MRI was conducted in 58 participants, subsequently grouped into absence of MRI-detectable lipid in carotid plaques (*n* = 31; Group 3) or presence of plaque lipid (*n* = 27; Group 4).

Normal distribution of each data subset was assessed using graphical methods and the Kolmogorov–Smirnov test. Because most variables showed a skewed distribution, descriptive data are expressed as medians and interquartile ranges (continuous variables) or as absolute frequencies and percentages (categorical variables). Differences among groups in clinical and laboratory characteristics, treatment regimes, carotid outcomes and dietary data were assessed by the chi-square test or Kruskal–Wallis tests, as appropriate. Spearman′s correlation coefficient was used to study the association between the calculated dietary intake of EPA + DHA and the RBC proportion of EPA in the overall population. Given that the RBC proportions of all tested fatty acids did not follow a normal distribution, these variables were processed by a logarithmic transformation in the regression analysis. Analysis of variance (ANOVA) was used to investigate differences regarding selected RBC fatty acids among groups differing in degree of carotid atherosclerosis. In addition, multiple linear regression analysis was used to study the association between EPA and imaging outcomes, including ultrasound-assessed maximum ICA-IMT and maximum IMT of all carotid territories (Groups 1 + 2 + 3 + 4, *n* = 161); ultrasound-assessed plaque burden (Groups 2 + 3 + 4, *n* = 123); MRI-assessed normalized wall index (Groups 3 + 4, *n* = 58); and MRI-assessed plaque lipid volume (Group 4, *n* = 27). This included an unadjusted model and a second model adjusted for age, gender, diabetes (yes/no), treatment with statins (yes/no), and ever smoking (yes/no) as potential confounders. Standard diagnostic checks on the residuals from the fitted models showed no evidence of any failure of the assumption of normality and homogeneity of the residual variance. Alternatively, we replaced EPA with DHA, omega-3 index, ALA, and arachidonic acid in all models. Statistical significance was set at the *P* < 0.05 level in all cases. Analyses were done using SPSS statistical software, version 16.0 (IBM Corp., Armonk, NY, USA).

## 3. Results

Table 1 shows clinical characteristics, treatment regimes and carotid imaging variables in the overall study population and in groups according to the severity of carotid atherosclerosis. Table 2 displays information regarding consumption of seafood products and derived EPA + DHA intake. None of the participating subjects reported consumption of omega-3 supplements. In the overall sample, the Spearman′s correlation coefficient between calculated intake of EPA + DHA and EPA ratio in RBC was 0.366 (*P* < 0.001). The omega-3 index was above 8% (the proposed low-risk cutoff for cardiovascular risk [17]) in 29.8% of the study group, and below 4% (highest-risk cutoff) in 3.1%.

**Figure 2:**
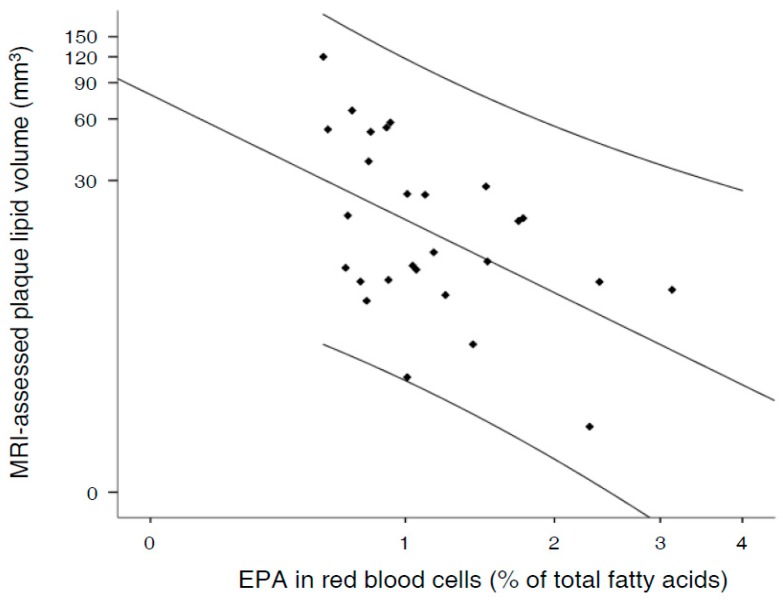
Scatter plot of the red blood cell proportion of EPA and plaque lipid core burden.

RBC proportions of EPA and other omega-3 fatty acids are provided in Table 3. No significant differences were observed among groups for RBC proportions of EPA (*P* = 0.697), DHA (*P* = 0.935), the omega-3 index (*P* = 0.631), ALA (*P* = 0.409) or arachidonic acid (*P* = 0.224). Coefficients of univariate associations between RBC EPA and carotid outcomes were 0.038 for maximum ICA-IMT (*P* = 0.656; *n* = 161); 0.090 for maximum IMT of all territories (*P* = 0.344, *n* = 161); 0.668 for plaque burden (*P* = 0.365; *n* = 123); and −0.002 for normalized wall index (*P* = 0.912; *n* = 58). Statistical significance was only observed for the association between EPA and MRI-assessed plaque lipid volume (univariate model; regression coefficient: −32.164; *P* = 0.006; *n* = 27), which is plotted in Figure 2. Significance was maintained after the inclusion of age, gender, and other cardiovascular risk factors as confounders in the regression model (multivariate model; regression coefficient: −31.300; 95% confidence interval: −57.685 to −4.915; *P* = 0.022) (Table 4). No significant associations were found for DHA, the omega-3 index, ALA, or arachidonic acid (data not shown).

## 4. Discussion

Here we report that in subjects at high vascular risk with MRI-detectable lipid core in carotid plaques, an increasing proportion of EPA in RBC relates inversely to plaque lipid burden, a well-known feature of unstable plaques. The novelty of the results is that this association is found in the context of the participants′ customary diet, without supplementation with either pure EPA or fish oil.

There is increasing interest in understanding how dietary habits contribute to the primary prevention of CHD, particularly in subjects at high vascular risk. In the present work, we focused on a valid and objective biomarker of long-term EPA intake in relation to carotid atherosclerosis. We assessed features of carotid plaques that are indicative of advanced yet subclinical atherosclerotic disease. These included ultrasound-assessed plaque burden; if large plaques were detected, MRI-assessed normalized wall index; and when a lipid core was present, plaque lipid volume. In addition to these variables, ultrasound assessment of ICA-IMT, which appears to be a better surrogate marker of future cardiovascular events than IMT at other carotid segments [18], allowed us to also include a subset of subjects without plaques. Interestingly, no differences in the proportion of EPA in RBCs were found across the spectrum of atherosclerotic disease, RBC EPA being solely related to a lower burden of atherosclerosis (plaque lipid volume) in subjects at the most advanced stage of the disease.

Our findings are in line with the suggestive evidence that EPA may not prevent plaque formation when significant risk factors are present, but could play a role in attenuating their vulnerability to rupture. Concurring with this notion, in a randomized trial conducted in patients awaiting carotid endarterectomy, retrieved plaques from subjects supplemented with EPA for several weeks before surgery showed increased stability in association with EPA enrichment compared to plaques from subjects receiving placebo [19]. Since CHD can be precipitated by inflammatory events occurring in advanced atheromatous plaques, it is plausible that stabilization of atheroma plaques underlies the significant reduction in major coronary events and non-fatal CHD after EPA supplementation observed in the Japan Eicosapentaenoic acid Lipid Intervention Study (JELIS) trial, conducted in Japanese patients with hypercholesterolemia [20], presumedly at high risk of advanced atheroma plaques. Such an effect is mechanistically supported by changes in the cell membrane by dietary EPA. They include direct changes in membrane lipid dynamics and structural organization [21] and, by virtue of displacement of the omega-6 arachidonic acid (C20:4*n*-6), changes in the production of eicosanoids and other lipid mediators by the cyclooxygenase, lipoxygenase, and cytochrome P450 pathways. Whereas arachidonic acid is a substrate for pro-inflammatory eicosanoids, the release of EPA from cell membranes leads to the generation of anti-inflammatory lipid mediators [22], thereby reducing the inflammatory milieu of advanced plaques [23]. In this regard, an EPA-derived lipid mediator, resolvin E1, has recently been found to promote a lower proportion of severe lesions in an animal model of advanced atherosclerosis [24].

Arachidonic acid has a marginal presence in dietary fats, hence, rather than reducing consumption of its parent foods (poultry and eggs), the best strategy to displace this fatty acid from plasma cell membranes is increasing EPA intake. In spite of a substantial westernization of dietary habits in the last decades in Spain, the supply of seafood (and therefore EPA) is still quite high [25]. Interestingly, the incidence of CHD events is delayed to older ages in Spain [26], where the prevalence of angina is similar to that reported in Western countries with much higher CHD rates [27], and in a forensic study the coronary arteries of young Spanish men frequently had stable plaques and lacked vulnerable plaques [28]. Taken together, these observations strongly suggest a slower evolution of coronary atherosclerosis in Spain. It is possible to surmise that high intakes of EPA, by attenuating plaque vulnerability to rupture, might explain in part the Spanish paradox of low CHD rates [29] in face of a high burden of cardiovascular risk factors [30].

Our study has limitations, such as small sample size, the cross-sectional nature and the uncertain generalization of our findings to younger individuals. In addition, the validity of using fatty acid composition in relative percentages and/or absolute amounts in blood components is a matter of debate, particularly regarding arachidonic acid [31], involved in the inflammatory response. However, data were expressed as percentages in a recent systematic review that collated 298 studies reporting the circulating EPA and DHA from healthy adults across the globe [32]. The study also has strengths, such as the use of state-of-the-art MRI technology to evaluate plaque composition, the use of an objective and stable biomarker of long-term fatty acid intake (reinforced by the significant association with calculated intake of EPA + DHA), and the adjustment for well-known confounders in multivariable analyses.

In conclusion, we report that in older individuals with asymptomatic carotid plaques not receiving omega-3 supplements, increasing proportions of EPA in RBCs relate to lower plaque lipid content, a surrogate of increased plaque stability. No significant differences in seafood consumption and its derived EPA + DHA were found among groups according to the severity of carotid atherosclerosis. We provide novel evidence contributing to the notion suggestive that regular fish consumption may not prevent plaque formation when significant risk factors are present, but could attenuate plaque vulnerability to rupture, reinforcing the notion of fish as a cost-effective strategy to prevent or at least delay coronary atherosclerosis, as recommended by guidelines of scientific societies.

## Figures and Tables

**Table 1:** Participants’ clinical and laboratory characteristics, treatment regimes and carotid outcomes.

Variable	Whole Sample (*n* = 161)	Absence of Plaque–Group 1 (*n* = 38)	Plaque <2 mm–Group 2 (*n* = 65)	Plaque ≥2 mm, Absence of Lipid-Rich Necrotic Core–Group 3 (*n* = 31)	Plaque ≥2 mm, Presence of Lipid-Rich Necrotic Core–Group 4 (*n* = 27)	*P* *
Male, no-%	83 (51.6)	16 (42.1)	24 (36.9)	18 (58.1)	25 (92.6)	<0.001
Age, years	65 (61 to 70)	62 (60 to 66) ^a^	65 (62 to 71) ^b^	65 (60 to 70) ^b^	65 (63 to 72) ^b^	0.027
Family history of early-onset CHD, no-%	73 (45.3)	19 (50.0)	36 (55.4)	10 (32.3)	8 (29.6)	0.007
Weight, kg	77.3 (69.2 to 86.3)	76.4 (69.2 to 84.3)	75 (66.5 to 87.3)	76.1 (68.8 to 89.0)	79.6 (72.6 to 86.4)	0.623
Body mass index, kg/m^2^	29.3 (27.15 to 31.9)	28.5 (26.9 to 32.78)	29.4 (27.6 to 32.4)	30.2 (27.4 to 31.7)	28.9 (27.1 to 31.0)	0.747
Waist circumference, cm	101 (96 to 107)	98 (94 to 110)	100 (96 to 105)	101 (96 to 108)	103 (98 to 107)	0.724
Current smoker, no-%	30 (18.6)	6 (15.8)	8 (12.3)	8 (25.8)	8 (29.6)	0.163
Diabetes, no-%	71 (44.1)	13 (34.2)	27 (41.5)	16 (51.6)	15 (55.6)	0.281
Hypertension, no-%	119 (73.9)	26 (68.4)	48 (73.8)	24 (77.4)	21 (77.8)	0.801
Dyslipidemia, no-%	112 (69.6)	23 (60.5)	48 (73.8)	23 (74.2)	18 (66.7)	0.484
Fasting glucose, mg/dL	107 (96 to 141)	102 (91 to 136)	107 (96 to 139)	115 (99 to 137)	121 (98 to 149)	0.221
Total cholesterol, mg/dL	211 (185 to 238)	208 (178 to 236)	214 (188 to 248)	205 (181 to 234)	212 (184 to 237)	0.388
LDL cholesterol, mg/dL	134 (108 to 154)	131 (103 to 155)	142 (115 to 159)	126 (111 to 143)	136 (99 to 151)	0.369
HDL cholesterol, mg/dL	50 (43 to 58)	52 (46 to 63) a	54 (44 to 60) a	45 (39 to 55) b	47 (39 to 49) b	0.001
Triglycerides, mg/dL	115 (86 to 161)	113 (76 to 153)	107 (79 to 158)	136 (106 to 179)	120 (99 to 176)	0.162
Use of statins, no-%	69 (49.2)	13 (34.2)	29 (44.6)	13 (41.9)	14 (51.9)	0.543
Use of antihypertensive agents, no-%	104 (64.6)	24 (63.2)	40 (61.5)	22 (71.0)	18 (66.7)	0.825
Use of oral antidiabetic agents, no-%	42 (26.1)	9 (23.7)	14 (21.5)	11 (35.5)	8 (29.6)	0.492
Maximum ICA-IMT, mm	--	0.75 (0.65 to 0.91) ^a^	0.93 (0.77 to 1.15) ^b^	1.11 (0.83 to 1.47) ^c^	1.14 (0.93 to 1.98) ^c^	<0.001
Plaque burden, mm	--	--	1.98 (1.40 to 3.12) ^a^	4.89 (3.30 to 7.58) ^b^	7.00 (4.71 to 11.65) ^c^	<0.001
Normalized wall index	--	--	--	0.47 (0.43 to 0.50)	0.50 (0.44 to 0.55)	0.086
Lipid-rich necrotic core, mm^3^	--	--	--	--	13.05 (9.14 to 37.27)	--

Values are expressed as medians (interquartile ranges), unless otherwise stated. CHD, coronary heart disease; LDL, low-density lipoprotein; HDL, high-density lipoprotein; ICA-IMT, intima-media thickness in internal carotid artery. * Comparisons among groups according to the degree of atherosclerosis. *P* value obtained by chi-square test and Kruskal–Wallis test, as appropriate. Medians with different superscript letters are significantly different (Mann–Whitney test).

**Table 2:** Consumption of seafood products and their associated EPA + DHA content.

Variable (g/Day)	Whole Sample (*n* = 161)	Absence of Plaque–Group 1 (*n* = 38)	Plaque <2 mm–Group 2 (*n* = 65)	Plaque ≥2 mm, Absence of Lipid-Rich Necrotic Core–Group 3 (*n* = 31)	Plaque ≥2 mm, Presence of Lipid-Rich Necrotic Core–Group 4 (*n* = 27)	*P* *
Total seafood ^†^	116 (79 to 145)	115 (76 to 139)	117 (86 to 152)	120 (75 to 145)	107 (67 to 135)	0.419
Fatty fish	19 (9 to 56)	19 (9 to 56)	19 (9 to 56)	19 (9 to 56)	19 (9 to 56)	0.643
Lean fish	64 (21 to 64)	64 (10 to 64)	64 (21 to 64)	64 (21 to 64)	64 (10 to 64)	0.571
Dietary EPA + DHA	0.81 (0.54 to 1.35)	0.86 (0.56 to 1.39)	0.84 (0.58 to 1.37)	0.76 (0.54 to 1.30)	0.71 (0.50 to 1.29)	0.612

Values are expressed as medians (interquartile range). EPA, eicosapentaenoic acid; DHA, docosahexaenoic acid. * Comparisons among groups according to the degree of atherosclerosis. *P* value obtained by Kruskal–Wallis test, as appropriate. ^†^ Including fatty fish, lean fish, mollusks, shrimp, prawn and crayfish, octopus and squid.

**Table 3:** Red blood cell proportions of selected fatty acids.

Fatty Acid (% of Total Fatty Acids)	Whole Sample (*n* = 161)	Absence of Plaque–Group 1 (*n* = 38)	Plaque <2 mm–Group 2 (*n* = 65)	Plaque ≥2 mm, Absence of Lipid-Rich Necrotic Core–Group 3 (*n* = 31)	Plaque ≥2 mm, Presence of Lipid-Rich Necrotic Core–Group 4 (*n* = 27)	*P* *
C18:3*n*-3 (alpha-linolenic acid)	0.17 (0.12 to 0.30)	0.20 (0.13 to 0.30)	0.17 (0.12 to 0.28)	0.20 (0.12 to 0.43)	0.14 (0.12 to 0.21)	0.409
C20:5*n*-3 (eicosapentaenoic acid)	1.00 (0.76 to 1.37)	1.04 (0.75 to 1.47)	1.00 (0.78 to 1.20)	0.87 (0.71 to 1.39)	1.01 (0.80 to 1.49)	0.697
C22:6*n*-3 (docosahexaenoic acid)	6.15 (5.17 to 6.95)	5.64 (5.18 to 6.80)	6.20 (5.12 to 7.02)	5.93 (4.88 to 7.01)	6.27 (4.94 to 6.94)	0.935
Omega-3 index (C20:5*n*-3 + C22:6*n*-3)	7.22 (6.11 to 8.23)	7.11 (5.99 to 8.16)	7.29 (6.07 to 8.14)	7.04 (5.75 to 8.22)	7.22 (6.89 to 8.77)	0.631
C20:4*n*-6 (arachidonic acid)	16.37 (14.50 to 18.51)	16.05 (14.55 to 17.32)	16.89 (14.40 to 18.71)	15.67 (13.92 to 18.60)	15.94 (14.90 to 18.09)	0.224

Data expressed as medians (interquartile ranges). * Obtained by ANOVA after logarithmic transformation.

**Table 4:** Independent determinants of plaque lipid core burden by multiple regression analysis.

Independent Variable	B (95% Confidence Interval)	*β*	*p*	Adjusted R^2^
Constant	6.284 (−119.057 to 131.625)		0.918	0.318
C20:5*n*-3 (log transformed)	−31.300 (−57.685 to −4.915)	−0.500	0.022	
Sex, female	−19.637 (−62.570 to 23.296)	−0.197	0.351	
Age, year	0.325 (−1.584 to 2.234)	0.078	0.726	
Diabetes at baseline, yes	7.177 (−14.161 to 28.515)	0.136	0.491	
Ever smoking at baseline, yes	−1.893 (−27.989 to 24.204)	−0.030	0.881	
Use of statins at baseline, yes	−1.007 (−22.761 to 20.747)	−0.019	0.924	
